# Quantitative Diagnosis of Colorectal Polyps by Spectral Domain Optical Coherence Tomography

**DOI:** 10.1155/2014/570629

**Published:** 2014-04-09

**Authors:** Chen Wang, Qinqin Zhang, Xiaojing Wu, Tao Tang, Hong Liu, S. W. Zhu, Bruce Z. Gao, X.-C. Yuan

**Affiliations:** ^1^Institute of Modern Optics, Key Laboratory of Optical Information Science & Technology, Ministry of Education of China, Nankai University, Tianjin 300071, China; ^2^Tianjin Union Medicine Centre, Tianjin 300121, China; ^3^Department of Bioengineering and COMSET, Clemson University, Clemson, SC 29634, USA; ^4^Institute of Micro & Nano Optics, College of Optoelectronic Engineering, Shenzhen University, Shenzhen 518060, China

## Abstract

The principal aim of this study is to investigate the scattering coefficient of colorectal polyp tissues using an optical coherence tomography (OCT) technique. It combines the existing scattering coefficient model and spectral domain OCT to achieve method of early diagnosis of colorectal polyp in hospitals. Seventeen patients were studied, and a total of 1456 data points were extracted by curve-fitting the OCT signals into a confocal single-backscattering model. The results show that the mean scattering coefficient value for colorectal polyps is 1.91 mm^−1^ (std: ±0.54 mm^−1^), which is between the values for normal and malignant tissues. In addition, we studied the difference between adenomatous polyps (*n* = 15) and inflammatory polyps (*n* = 2) quantitatively and found that the adenomatous tissues had lower scattering coefficients than the inflammatory ones. The quantitative measurements confirmed that OCT can be used in primary diagnosis to compensate for the deficiencies in methods of pathological diagnosis, with a great potential for early diagnosis of tissues.

## 1. Introduction

Colorectal cancer, the third leading cause of cancer-related morbidity and mortality worldwide, afflicts more than one million patients annually, with an annual mortality rate of 25% [1]. Western countries like the USA have for decades used early screening and monitoring of colorectal cancer nationwide for early diagnosis and treatment. Fortunately, because colorectal cancer develops slowly via polyps, a process usually requiring more than 10 years, it can be detected early and so is among the most preventable cancers [2–4]. Colorectal polyps are redundant growths on the lining of the colon or rectum; usually benign, they are thought to be precursor lesions of many colorectal cancers. Typically, polyps become more common with age, and larger ones have a greater risk of being cancerous than smaller ones. Besides, multiple polyposis is more likely to become cancerous. People with a family history of colon cancer or colon polyps may have a big risk. Removal of polyps by endoscopic resection is the preferred treatment, with sections being prepared for pathological diagnosis. However, owing to complex reasons such as the shape, location, and size, some polyps may be ablated during operation. Moreover, for patients with multiple polyps, each polyp's location cannot be identified after resection. Thus, there is an urgent need to develop an in situ diagnostic method that can be used with resection.

Spectral domain optical coherence tomography (SDOCT), a noninvasive imaging technology that provides an internal microstructural image with high resolution [5–7], is widely used in imaging microstructural biological features in the eyes, nerve fibers, brain, and so forth. [8–12]. In recent years, a method of quantitatively analyzing OCT images has been developed that evaluates tissue features by calculating scattering coefficient ([13–19]). Using this method, we can achieve quantified diagnoses which are more objective and convenient.

In previous work, we used this method to quantify differences between normal and malignant rectal tissues [20]. Polyp is a form between normal and malignance in pathology. We hypothesize that the scattering coefficient of colorectal polyp tissues obtained from OCT can reflect the level of their malignancy. To test our hypothesis, we explored the scattering coefficient of colorectal polyps and made a contrast to the work we had done before. Besides, we explored the scattering coefficient of different types of tissues and concluded that our system is able to make a distinction between adenomatous polyps and inflammatory polyps, which may have further use to evaluate the risk of patients to develop cancers.

## 2. Methods

### 2.1. The OCT System

**Figure 1:**
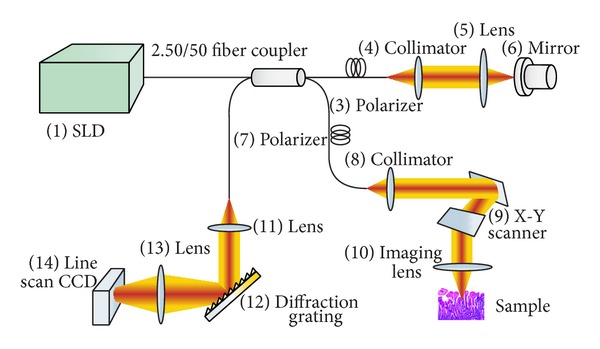
Schematic of the SDOCT system.

This study used a two-part setup: a fiber-based Michelson interferometer and a spectrometer. The interferometer is composed of a light source and the sample and detection arms. The light source is a superluminescent diode (S-840-B-I-20) with a central wavelength of 840 nm and a bandwidth (FWHM) of 50 nm. The beam from the light source is separated by a 50 : 50 fiber coupler into two parts, which are, respectively, directed into the reference and sample arms. To generate an interference signal with high contrast, the polarization of each beam is controlled by polarizers to be identical. The two beams reflected from the reference mirror and the sample are recombined and interfere with each other. The interfering beam is subsequently analyzed by a spectrometer, which includes a collimating lens, a diffraction grating (1200 lines mm^−1^), an achromatic focusing lens, and a line scanning CCD (e2v AViiVA SM2 CL, 4096 × 1, and 10 *μ*m). After calibration using a standard light source, the spectrometer's resolution was estimated to be 0.0455 nm, which made the OCT detecting depth approximately 2.58 mm. The system's axial resolution was theoretically and experimentally determined to be approximately 6.2 *μ*m and the lateral resolution approximately 9.5 *μ*m.  Figure 1 shows a schematic of the system.

### 2.2. The Models for OCT Measurement

Two models are widely used to describe OCT signals: the single-backscattering model and the multiple-scattering model ([13, 16, 20, 21]). The former is suitable for weakly scattering media and the latter for highly scattering media. The single-backscattering model was used for this study. Considering the confocal properties, the model is described as follows:

(1) $$\begin{array}{c} I\left(z\right)\propto \frac{\exp\left(-2\mu_{t}z\right)}{{\left(\left(z-z_{\text{cf}}\right)/z_{R}\right)}^{2}+1}, \end{array}$$

where *z*_cf_ is the focal length and *z*_*R*_ is the apparent Rayleigh length [20]. Based on this model, the reflective coefficient can be obtained from an OCT measurement.

### 2.3. Experiment and Results

Polyp tissues from 17 patients aged 10–79 years were studied; detailed patient information is presented in Table 1. The study protocol was approved by the ethical committee of Tianjin Union Medicine Centre and signed informed consent was obtained from all patients. Based on the dynamic scanning range of our scanning mirrors, our tissue samples were larger than 1 mm, and multiple regions were scanned for each sample.

Fresh polyp samples were placed on a sample platform immediately after retrieval. To separate the mirror image intrinsic to Fourier transform-based image processing, the focal plane of the object lens, which was set at the sample surface, was placed below the zero-delay line of the OCT system. Because polyp size was diverse, 5–20 OCT images (B-scans) were acquired of each tissue sample. Every B-scan consisted of 500 A-scans, and each A-scan consisted of 4096 pixels. One image could be completed in about 15 seconds. In the image processing, 100 adjacent A-scans were selected as the region of interest (ROI) to calculate an averaged OCT signal *I*(*z*) corresponding to each depth (*z*); then, the scattering coefficient, *μ*_*s*_, was estimated by fitting the averaged *I*(*z*) to the model described by (1). To avoid interface effects, fitting started approximately 20 *μ*m below the tissue surface. Three-to-five ROIs were chosen in every sample to obtain three-to-five scattering coefficients. We obtained approximately 1500 scattering coefficient values in total. After OCT imaging, the samples were fixed in formalin, embedded in paraffin, sectioned into 5 *μ*m samples, and stained with hematoxylin and eosin (H&E).

**Figure 2:**
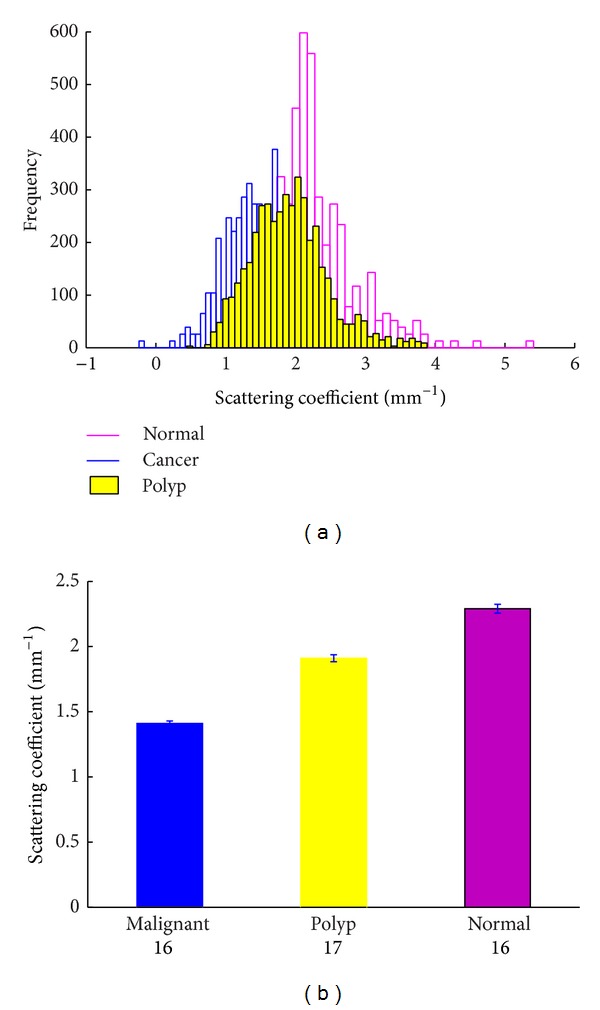
(a) Normalized histograms of normal, polyp, and malignant tissue scattering coefficients; (b) comparison of *μ*_*s*_ obtained from each tissue group. The error bars represent 95% confidence intervals of the extracted fit parameter. The numbers below each bar represent the number in the sample.

All scattering coefficient measurement data were grouped in terms of their value for statistical analysis.  Table 2 lists the normalized percentages of each range of scattering coefficient. Statistical analysis showed that the scattering coefficient ranged from 0.44 to 3.88 mm^−1^ with a mean value of 1.91 mm^−1^ (std = ±0.54). In previous work, we assessed 16 cases of malignant and normal colorectal tissues and found that the scattering coefficient of malignant tissues ranged from 0.25 to 2.69 mm^−1^ with a mean value of 1.41 mm^−1^ (std = ±0.18), and the values of normal tissues ranged from 1.09 to 5.41 mm^−1^ with a mean value of 2.29 mm^−1^ (std = ±0.32) [20]. The polyp scattering coefficient obtained in this study was between the values obtained from normal and malignant tissues as indicated by the comparison of histograms in Figure 2(a) and the bar graph in Figure 2(b). The standard deviation of the polyp group is relatively large because the polyp tissues are composed of various components including normal mucosal tissues, adenoma tissues, inflammation tissues, and malignant tissues. In the scanning process of the experiment, we could not differentiate the boundary of mucosa and lesion tissues, so the scattering coefficients of the polyps summed up might cover a relatively large range. The error bars in Figure 2(b) represent the 95% confidence intervals of the fitted *μ*_*s*_.

**Figure 3:**
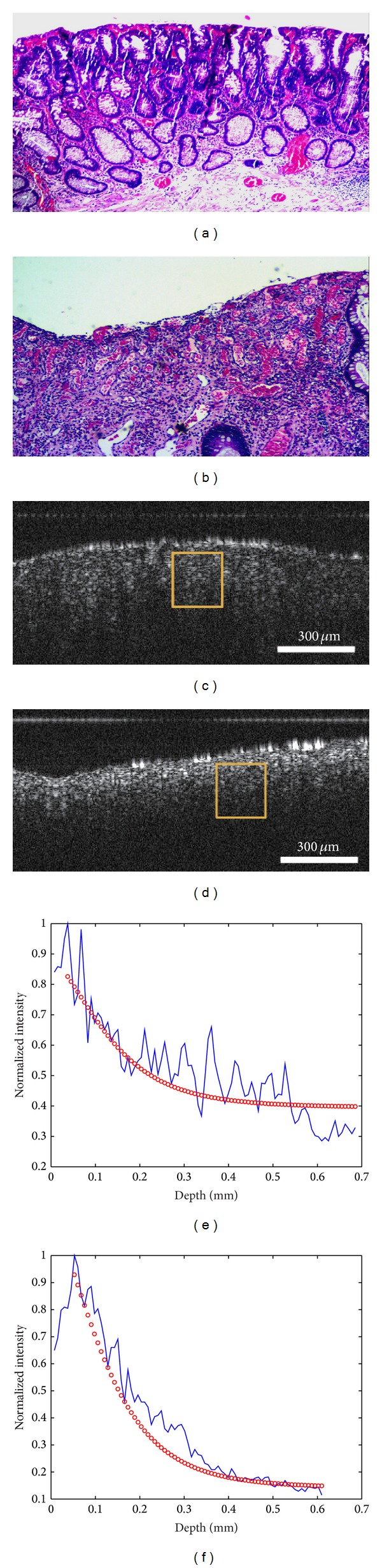
Representative (a) adenomatous and (b) inflammatory polyp tissue. (a) and (b) Pathological images; (c) and (d) OCT images. The curve fitting of images (a) and (b) is shown in (e) and (f).

**Figure 4:**
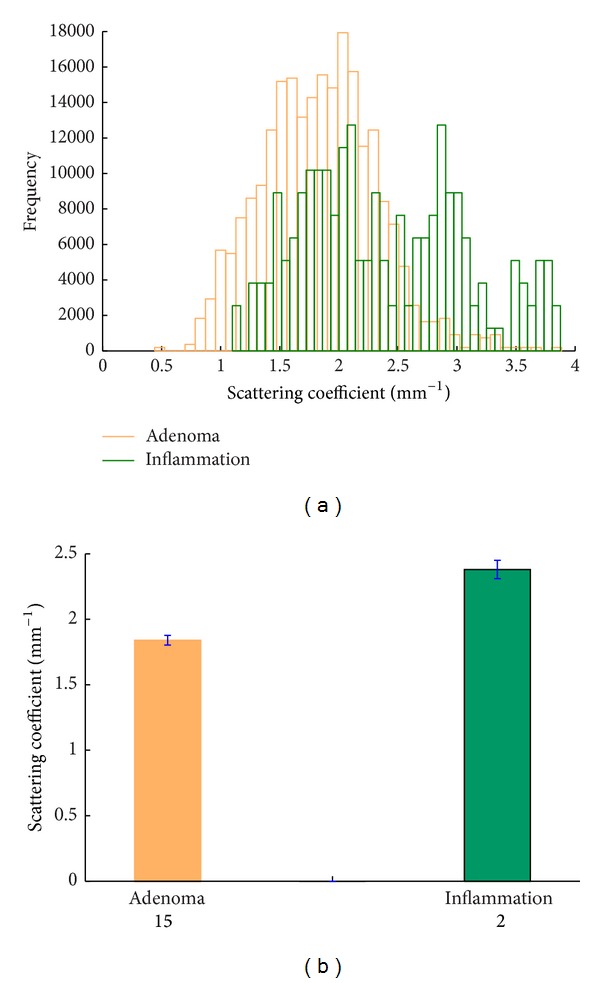
(a) Normalized histograms of scattering coefficients of adenomatous and inflammatory polyp tissues; (b) comparison of *μ*_*s*_ obtained from each tissue group. The error bars represent 95% confidence intervals of the extracted fit parameter. The numbers below each bar represent the number in each sample.

Colorectal polyps are often classified according to their behavior and etiology as hyperplastic, neoplastic, hamartomatous, and inflammatory. As shown in Table 1, 15 of the 17 patients in this experiment had adenomatous polyps while 2 had inflammatory polyps.  Figure 3 shows the pathological and OCT images of an adenomatous polyp and an inflammatory polyp.  Figure 3(a) is a pathological image of an adenomatous polyp showing tubular structure and multiple crypts on its surface, which form a regular array.  Figure 3(b) is a histological image of inflammatory polyps showing a large amount of inflammatory granulation accompanied with inflammatory cell infiltration. It also demonstrates hemangiectasia and congestion pathology. The corresponding OCT images (Figures 3(c) and 3(d)) demonstrate that the surface reflection of the inflammatory tissue is greater than that of the adenomatous tissue and the detection depth for the adenomatous tissue is greater than that of the inflammatory tissue. These differences can be attributed to the structures of the two tissue types. Figures 3(e) and 3(f) show examples of curve fitting for estimating the corresponding scattering coefficients. The fitting results are shown in Figure 4(a) in a histogrammatic format and in Figure 4(b) in a bar graph format. The mean value of the adenomatous polyps is 1.84 (std = ±0.68) and that of the inflammatory polyps is 2.38 (std = ±0.48). The 95% confidence intervals were also calculated for more insightful estimates, which were shown in Figure 4(b) as error bars. The two values are significantly different (*P* < 0.001) based on Student's *t-test*. Clinically, inflammatory polyps are associated with conditions such as ulcerative colitis and Crohn's disease and have little risk of becoming cancerous. Adenomatous polyps are benign neoplasms with a higher risk of becoming cancerous. Our study demonstrated that inflammatory polyps have relatively higher scattering coefficients, which are similar to those of normal tissue, and adenomatous polyps have lower scattering coefficients, which are similar to those of malignant tissue. These results are consistent with pathological analysis. Thus OCT data have the potential to be used to diagnose polyps and make an evaluation of their malignant potential. Considering the feasibility of constructing an endoscopic OCT, the diagnosis can be performed in situ during colonoscopy. Further work should be conducted to evaluate distinguishing characteristics among OCT images of different polyp tissues, such as hamartomatous, hyperplastic, adenomatous, and inflammatory tissues and the sample size should be extended to ensure the accuracy of conclusion.

## 3. Summary

In this study, we investigated the optical scattering properties of colorectal polyp tissues using optical coherence tomography and demonstrated that the scattering coefficient of polyp tissues was between those of normal and malignant tissues. We also analyzed the difference between adenomatous polyps and inflammatory polyps and showed that the adenomatous tissue had a lower scattering coefficient similar to malignant tissue, and inflammatory tissues, similar to a normal tissue, had a higher scattering coefficient. These data demonstrated that the scattering coefficients obtained by the OCT technique may be used in diagnosis of polyp tissues, with significant potentials for preventing cancers.

The system reported here provided only ex vivo data. In future research, an endoscopic OCT system should be developed to provide quantitative in vivo diagnostic data, which is more attractive in clinical medicine.

## Figures and Tables

**Table 1:** Detailed patient information.

Patient number	Sex	Age	Admitting diagnosis (AD)	Operation information	Pathological diagnosis
191899	Female	63	Colon polyp	Endoscopic colon polyp resection	Tubular adenoma; high-level focal epithelial tumor lesions
192429	Female	61	Colon multiple polyp	Endoscopic colon polyp resection	Polypoid mucosal tissues; chronic inflammation
186807	Male	62	Sigmoid colon polyps	Endoscopic colon polyp resection	Tubular adenoma; chronic inflammation
194932	Male	74	Sigmoid colon polyps	Endoscopic colon polyp resection	Tubular adenoma
194934	Male	62	Colon multiple polyp	Endoscopic colon polyp resection	Tubular adenoma; interstitial inflammatory cell infiltration; focal organization pressing
198305	Male	62	Colon polyp	Endoscopic colon polyp resection	Tubular adenoma
198656	Male	47	Colorectal polyp	Endoscopic colorectal polyp resection	Tubular adenoma; glandular epithelium mild-to-moderate atypical hyperplasia
198720	Male	62	Colon polyp	Endoscopic colon polyp resection	Tubular adenoma; glandular epithelium mild-to-moderate atypical hyperplasia; a few muscularis mucosae
200397	Female	69	Colon multiple polyp	Endoscopic colon polyp resection	Tubular adenoma; glandular epithelium mild atypical hyperplasia; acute and chronic inflammation
203040	Male	72	Colon multiple polyp	Endoscopic colon polyp resection	Multiple tubular adenoma; polypoid intestinal mucosal tissue; superficial erosion; propria interstitial cell hyperplasia
204105	Female	58	Colon polyp	Endoscopic colon polyp resection	Tubular adenoma
205418	Male	60	Colon polyp	Endoscopic colon polyp resection	Tubular adenoma; brown polypoid tissues
146723	Male	79	Colon multiple polyp	Endoscopic colorectal polyp resection	Multiple tubular adenoma; glandular epithelium moderate atypical hyperplasia; focal severe atypical hyperplasia; growing actively
205810	Female	10	Rectal polyp	Endoscopic rectal polyp resection + APC	juvenile inflammatory polyp
204950	Male	68	Colon polyp	Endoscopic colorectal polyp resection	Tubular adenoma
206140	Female	67	Colon multiple polyp	Endoscopic colon polyp resection + APC	Tubular adenoma; glandular epithelium mild-to-moderate atypical hyperplasia
206425	Male	51	Colon multiple polyp	Endoscopic colon polyp resection + APC	Tubular adenoma; interstitial hemorrhage and focal epithelium mild-to-moderate atypical hyperplasia

**Table 2:** Normalized percentages of each range of scattering coefficient.

Range of scattering coefficient (mm^−1^)	Percentage
0.5	0.07%
1	2.82%
1.5	20.60%
2	35.37%
2.5	29.33%
3	7.83%
3.5	2.68%
4	1.30%
